# Computational discovery and annotation of conserved small open reading frames in fungal genomes

**DOI:** 10.1186/s12859-018-2550-2

**Published:** 2019-02-04

**Authors:** Shuhaila Mat-Sharani, Mohd Firdaus-Raih

**Affiliations:** 1 Centre for Frontier Sciences, Faculty of Science and Technology, Universiti Kebangsaan Malaysia, UKM, 43600 Bangi, Selangor Malaysia; 2 Malaysia Genome Institute, Ministry of Science, Technology & Innovation, Jalan Bangi, 43000 Kajang, Selangor Malaysia; 3 Institute of Systems Biology, Universiti Kebangsaan Malaysia, UKM, 43600 Bangi, Selangor Malaysia

**Keywords:** Small open Reading frames, sORFs, smORF, Conserved, Fungal

## Abstract

**Background:**

Small open reading frames (smORF/sORFs) that encode short protein sequences are often overlooked during the standard gene prediction process thus leading to many sORFs being left undiscovered and/or misannotated. For many genomes, a second round of sORF targeted gene prediction can complement the existing annotation. In this study, we specifically targeted the identification of ORFs encoding for 80 amino acid residues or less from 31 fungal genomes. We then compared the predicted sORFs and analysed those that are highly conserved among the genomes.

**Results:**

A first set of sORFs was identified from existing annotations that fitted the maximum of 80 residues criterion. A second set was predicted using parameters that specifically searched for ORF candidates of 80 codons or less in the exonic, intronic and intergenic sequences of the subject genomes. A total of 1986 conserved sORFs were predicted and characterized.

**Conclusions:**

It is evident that numerous open reading frames that could potentially encode for polypeptides consisting of 80 amino acid residues or less are overlooked during standard gene prediction and annotation. From our results, additional targeted reannotation of genomes is clearly able to complement standard genome annotation to identify sORFs. Due to the lack of, and limitations with experimental validation, we propose that a simple conservation analysis can provide an acceptable means of ensuring that the predicted sORFs are sufficiently clear of gene prediction artefacts.

**Electronic supplementary material:**

The online version of this article (10.1186/s12859-018-2550-2) contains supplementary material, which is available to authorized users.

## Background

Small open reading frames (smORF) are sequences that potentially encode for proteins but are shorter than other more commonly translated genomic DNA sequences [1]. Such protein sequences can theoretically range from a minimum of two to ~ 100 residues. Various values have been reported for what can be acceptable as the limits to be a small and functional protein. The problem of determining what constitutes the minimum number of codons to be considered as protein coding has been discussed since the earliest genome sequences for *Saccharomyces cerevisiae* were published [2, 3]. In addition to the term smORF, these sequences have also been referred to as short open reading frames (sORFs) and the proteins that they encode have at times been referred to as microproteins.

Despite the term sORF turning up only in more recent literature, the existence of genes that code for proteins of 150 residues and less have been known for more than three decades. Functional sORFs have been identified in a wide range of organisms from prokaryotes to humans. The Sda protein (46 residues) found in *Bacillus subtilis* is known to inhibit sporulation by preventing the activation of a required transcription factor [4, 5]. Proteins such as TAL (11 residues), found in *Drosophila melanogaster*, are known to be important for leg development [6, 7]. The Cg-1 protein (< 33 amino acids) is involved in controlling tomato-nematode interaction [8]. In *Homo sapiens*, the humanin (24 amino acids) protein is involved in mitochondria-nuclear retrograde signalling that controls apoptosis [9, 10]. Possibly the smallest ORF reported to date encodes a six residue polypeptide – MAGDIS; this ORF is referred to as the upstream open reading frame (uORF) in the mRNA of S-adenosylmethionine decarboxylase (AdoMetDC), a key enzyme in the polyamine biosynthesis pathway [11].

As genome sequencing capabilities steadily progressed from the late 90s, through the 2000s to the present, many studies have identified and annotated sORFs directly from genome sequence data [12]. Various reports of sORFs discovered from such efforts have been published such as in *Escherichia coli* (15–20 amino acids) [13]; in yeast *- Saccharomyces cerevisiae* (less than 100 amino acids) [12, 14]; in plants - *Arabidopsis thaliana* (100–150 amino aicds) [15] and *Bradyrhizobium japonicum* (less than 80 amino acids) [16]; in insects - *Drosophila* (less than 100 amino acids) [17]; in mouse (less than 100 amino acids) [18] and in human (less than 100 amino acids) [19]. More recently, Erpf and Fraser reviewed the diverse roles of sORF encoded peptides (less than 150 amino acids) in fungi [20].

Nevertheless, it has also been shown that many ORFs with lengths of 100 or less amino acids may have been missed during gene prediction from whole genome sequences because the gene prediction tools are tuned to ignore small genes perceived to be ‘junk’ or non-protein coding [21]. For example, the early genome annotations of *S. cerevisiae* had defined 100 residues and 150 residues as the minimum number to be encoded by an ORF thus in a way setting a parameter value for future gene predictions and annotation work [2, 3]. Perhaps as a consequence of such practices being integrated as part of standard gene prediction protocols, the number of sORFs that have been identified over the years has remained relatively small. Although the parameters for the gene prediction can be tweaked and changed in light of a better understanding regarding the existence of sORFs, the challenge of ascertaining that the annotated sORFs are indeed protein coding and not artefacts remains [17].

In this work, we have identified potential sORFs from fungal genomes by specifically repeating the gene prediction and annotation processes based on a residue length cutoff of 80 amino acids or less and specified the range of sORFs length distribution among homologs to avoid false positives. The cutoff of 80 residues was chosen as a simplistic means of selecting ORFs that were most likely to have been overlooked by previous gene predictions. The identification of 1986 putative predicted sORFs involved a large sequence dataset extracted from 31 fungal genome sequences with a total of 210,928 ORFs from existing gene prediction and annotation. The predicted sORFs were then compared to identify highly conserved examples within the fungal genomes dataset by adopting the assumption that such highly conserved sequences may code for common or even essential functions and are thus unlikely to be artefacts or randomly matched examples. This can potentially be a quick and inexpensive means of identifying subsets of sORFs that are classified as hypothetical proteins for experimental characterization.

## Results

### Identification of potential sORFs

The fungal genomes selected were required to have associated annotations for predicted genes thus limiting our dataset to only 31 genomes at the time the work was initiated. These annotations were utilized to identify 5255 sORFs genes that had already been identified in the original annotation to encode for a maximum of 80 amino acid residues. The ORF prediction process was then repeated for all 31 fungal genomes using the computer programs getorf [22, 23] and sORFfinder [24] as detailed in methods section. This process resulted in 16,156,945 sORFs identified by getorf and 902,110 found by sORFfinder. The results of both searches were overlapped to yield a consensus of 42,587 potential sORFs sequences encoding for 80 residues or less. The ORFs predicted by getorf with a cutoff of 240 nt were considered as genes that can either be a region that is free of STOP codons or a region that begins with a START codon and ends with a STOP codon [22, 23]. However, all the sORFs identified in this study have both START as well as STOP codons.

### Characterization of the fungal conserved sORFs

**Fig. 1:**
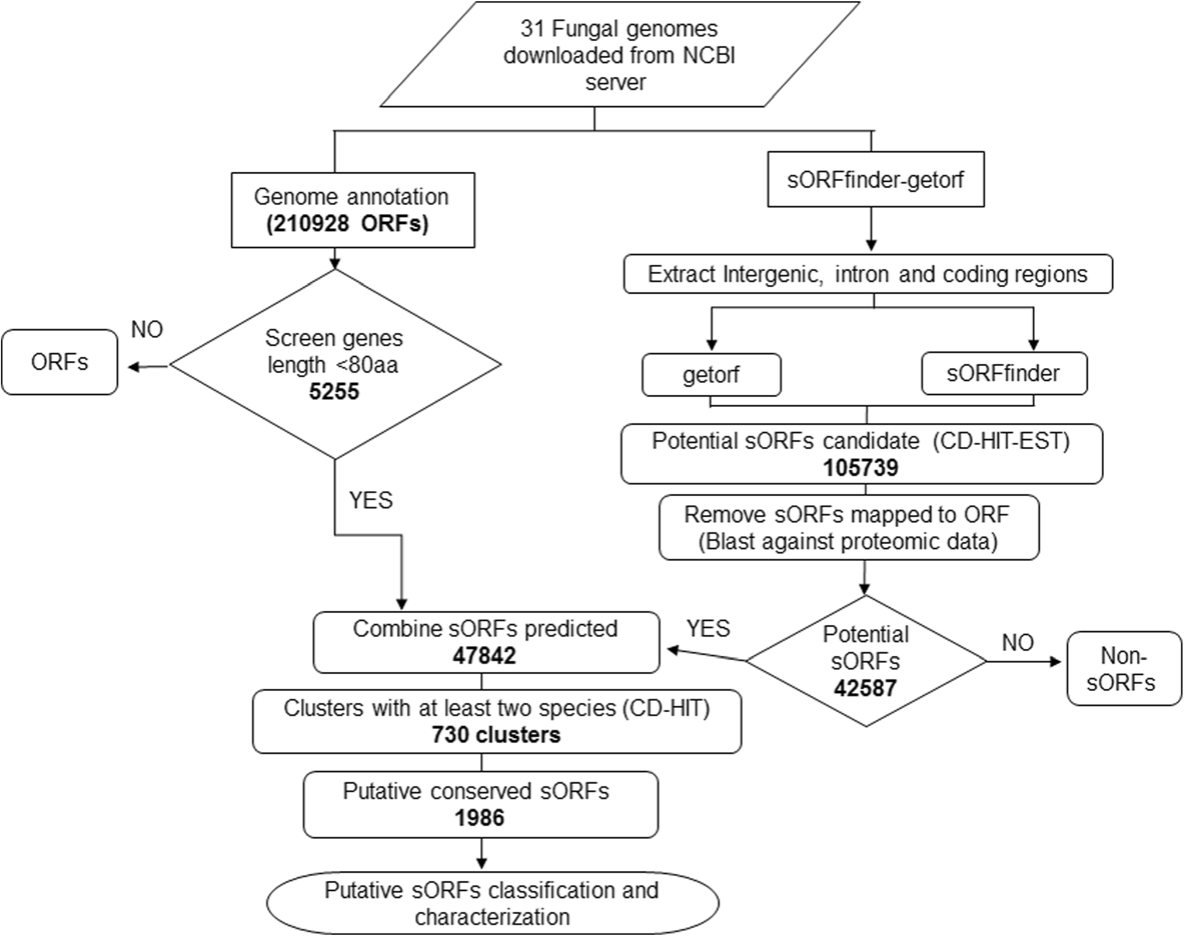
Research workflow

**Fig. 2:**
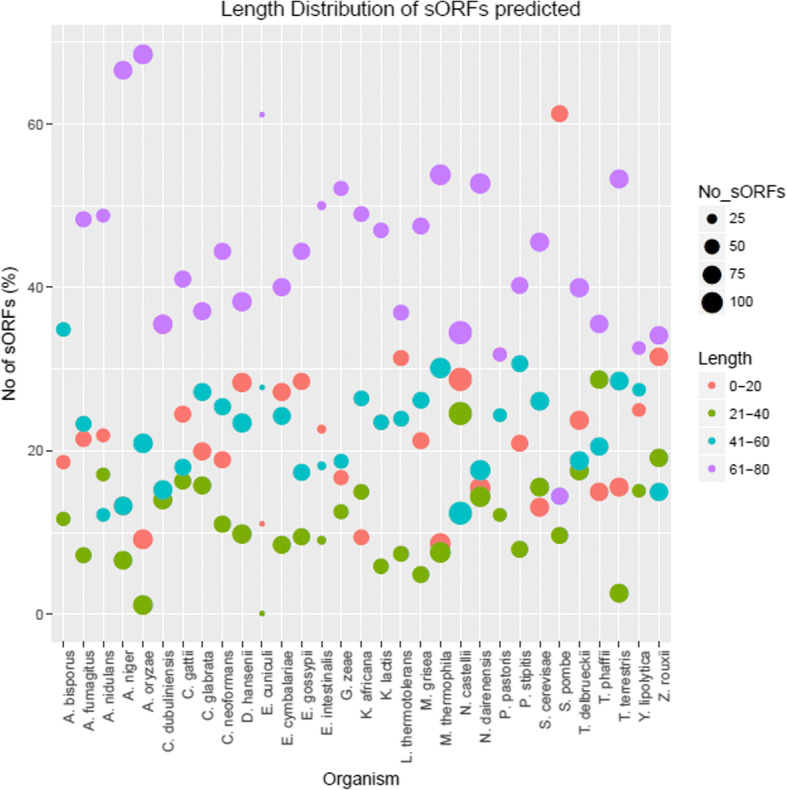
Distribution of sORFs according to length for all 31 fungal genomes

CD-HIT [25] clustering of the combined 47,842 potential sORFs at 70% sequence identity resulted in 730 sORFs clusters that comprised of 1986 sequences putatively conserved in at least two fungal species (Fig. 1). Four of the sORFs predicted have Kozak sequences based on an ORF integrity value that was derived by calculating their coding potential using CPC2 [26]. The majority of the sORFs predicted were in the under 40 residues range with the shortest sORF in this dataset being composed of only 11 amino acids (Fig. 2).

**Fig. 3:**
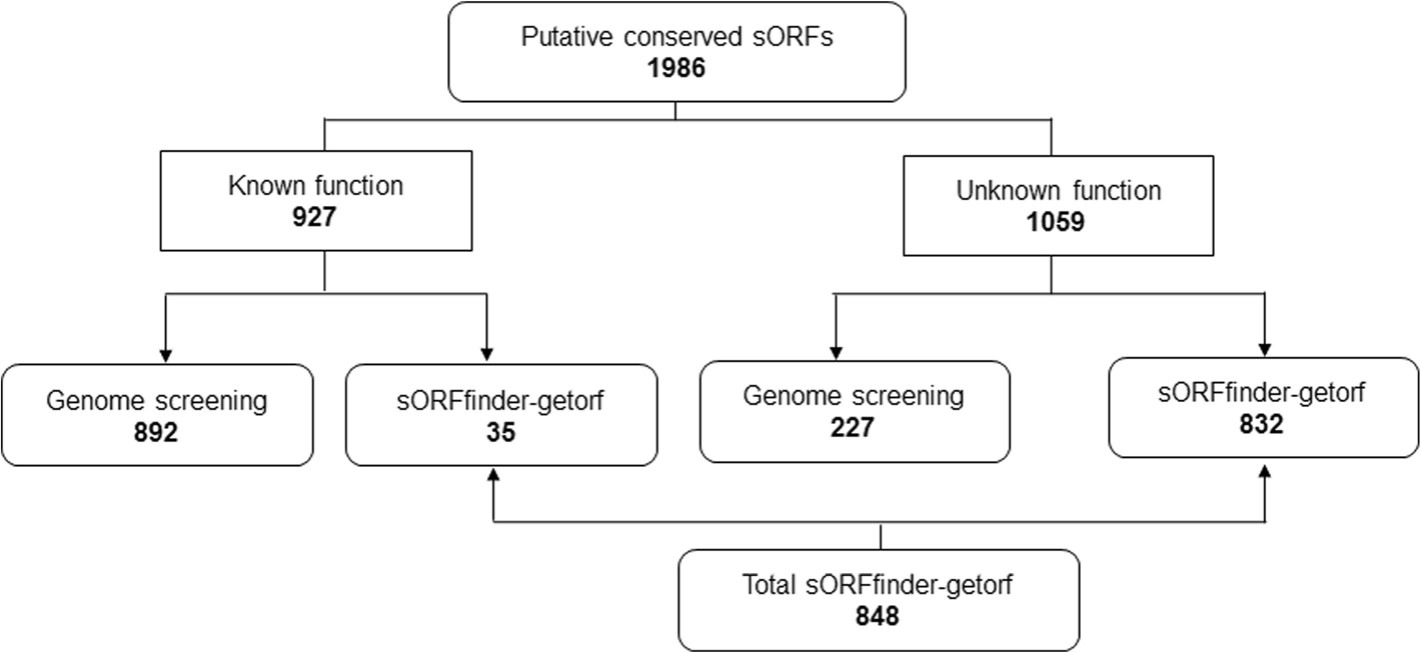
Distribution of total of conserved sORFs across fungal genomes

The clustering based on 70% similarity of the 47,842 potential sORFs resulted in a total of 1986 putative sORFs that are conserved within at least two fungal species (Additional file 1). Among the 1986 putatively conserved sORFs, 927 have homologs with known functions (35 from the purpose built sORF prediction process; 892 from existing genome annotations) (Fig. 3). The remainder 1059 putatively conserved sORFs have uncharacterized functions and can be further divided into two categories: the first set - 23 sORFs with homologs outside of the 31 fungal genomes; and the second set - 1036 sORFs with no detectable sequence homologs outside of the 31 fungal genomes. The latter set can thus be considered as fungi specific sORFs.

## Discussion

The standard gene prediction process may miss ORFs that encode for protein sequences of less than 100 residues [12, 27]. In order to address this, we carried out a two pronged approach using a dataset of 31 available fungal genomes to carry out: (i) identification of ORFs that have already been annotated to be below 80 residues in length and (ii) repeating the gene prediction process for each genome to specifically identify genes that encode for sORFs of 80 residues or less.

The bias of the parameter often used to predict genes that require a minimum of 100 codons may bypass the sequences in the intergenic spaces, especially when such regions are less than 100 residues in length, but yet they may actually encode for functional proteins of less than 80 residues. Intronic sequences may also hypothetically code for such sORFs. Therefore, these sequences were used as the target for sORF searches in this work. Predicted sORFs that were found to occur in multiple genomes were selected for further characterization. However, it was anticipated that such searches can return a large number of predicted genes, many of which could be artefacts of the search process itself. In order to address this, the pool of predicted sORFs were then compared to each other to find potentially homologous sequences within the predicted sORFs dataset. It is expected that such short sequences that are conserved in several genomes can be assumed to be of functional importance and thus not an artefact of the gene prediction process, especially more so if those sequences were also extracted from the intronic regions as was the case in this study.

At the time this work was initiated, 31 fungal genomes were selected because they had relatively complete genome sequences and had accompanying annotations. All the selected genomes were from the kingdom fungi and from various phyla including Ascomycota, Basidiomycota and Microsporidia (Table 1). Due to this diversity, we therefore believe that the workflow developed would be widely applicable for all fungi and possibly for other kingdoms as well.

**Table 1:** List of sORFs predicted from the whole genome and intergenic regions of fungal genomes

Microorganisms (yeast/fungi)	Phylum	Genome size (Mb)	Scaffolds(sc)/ Chromosomes	Intergenic region	Intron	ORF	sORFs from genome annotation	sORF from ab initio prediction						Total sORFs predicted
								GetORF		sORFfinder	Combined 1st & 2nd approached	sORFs match ORF homolog	sORFs ab initio predicted	
								Genome	Intergenic					
Agaricus bisporus	Basidiomycetes	30.78	29sc	10,606	50,859	10,450	575	423,332	113,990	902,110	2808	1952	856	1431
Aspergillus fumigatus	Ascomycetes	29.39	16	9916	18,630	9630	180	329,750	644,099	746,886	6665	5212	1453	1633
Aspergillus nidulans	Ascomycetes	29.83	17	9410	25,192	9410	83	376,689	533,546	288,159	3031	1803	1228	1311
Aspergillus niger	Ascomycetes	38.50	20sc	10,828	25,160	10,609	90	454,556	645,989	431,379	2950	1690	1260	1350
Aspergillus oryzae	Ascomycetes	37.91	27sc	12,937	29,686	12,818	172	508,537	639,035	361,674	3336	2042	1294	1466
Candida dubliniensis	Ascomycetes	14.62	8	5499	169	5213	45	180,147	249,542	181,108	4591	2046	2545	2590
Candida glabrata	Ascomycetes	12.34	14	6580	33,084	6575	159	153,883	203,269	290,815	2593	1271	1322	1481
Cryptococcus gattii	Basidiomycetes	18.37	14	6617	34,336	6475	94	250,564	331,357	263,216	2447	1864	583	677
*Cryptococcus neoformans*	Basidiomycetes	2.50	14	6658	479	6290	110	254,930	329,135	247,268	2266	1673	593	703
*Debaryomyces hansenii*	Ascomycetes	2.25	8	2029	32	1996	300	153,782	190,959	165,774	4179	2540	1639	1939
Encephalitozoon cuniculi	Microsporidia	2.22	11	1892	14	1833	13	28,102	33,715	53,062	554	396	158	171
Encephalitozoon intestinalis	Microsporidia	2.19	11	4853	239	4434	18	26,156	28,950	54,302	543	412	131	149
Eremothecium cymbalariae	Microsporidia	9.67	8	5356	276	4776	61	119,153	153,876	171,154	3384	1865	1519	1580
Eremothecium gossypii	Microsporidia	9.12	8	11,624	25,808	11,628	80	94,967	117,788	121,934	3529	2265	1264	1344
Gibberella zeae	Ascomycetes	38.05	11	5649	1040	5378	104	488,905	698,951	151,211	4487	2265	1832	1936
Kazachstania africana	Ascomycetes	11.13	12	5649	1040	5378	88	144,748	186,855	141,681	1894	1247	647	735
Kluyveromyces lactis	Ascomycetes	10.73	7	5412	182	5085	73	139,746	182,755	81,044	3006	1636	1370	1443
Lachancea thermotolerans	Ascomycetes	10.39	8	5498	284	5091	53	114,598	145,278	155,291	3134	1628	1506	1559
Magnaporthe grisea	Ascomycetes	40.30	8	14,210	25,265	14,014	1105	506,420	699,033	217,609	4466	2735	1731	2836
Myceliophthora thermophila	Ascomycetes	38.74	7	9294	15,500	9099	419	343,719	540,155	140,855	4738	2547	2191	2610
Naumovozyma castellii	Ascomycetes	11.22	10	5870	203	5592	102	147,551	187,871	187,462	5691	3430	2261	2363
Naumovozyma dairenensis	Ascomycetes	13.53	12	6057	177	5772	99	185,531	251,917	231,996	2841	1323	1518	1617
Pichia pastoris	Ascomycetes	9.60	4	5040	578	5040	89	112,299	138,917	61,604	4161	2872	1289	1378
Pichia stipitis	Ascomycetes	15.44	8	5816	2567	5816	61	186,356	259,185	79,673	2146	1008	1138	1199
*Saccharomyces cerevisiae*	Ascomycetes	12.16	17	6349	366	5906	224	153,187	200,252	398,979	3478	2196	1282	1506
Schizosaccharomyces pombe	Ascomycetes	12.59	4	6991	3793	5133	124	165,857	165,864	33,542	6027	3304	2723	2847
Tetrapisispora phaffii	Ascomycetes	12.12	16	5460	141	5250	89	153,895	206,397	825,322	2959	1410	1549	1638
Thielavia terrestris	Ascomycetes	36.91	6	9958	17,290	9802	72	296,185	443,166	163,032	3291	1966	1325	1397
Torulaspora delbrueckii	Ascomycetes	9.22	8	5176	203	4972	402	113,250	138,118	509,930	4521	3074	1447	1849
Yarrowia lipolytica	Ascomycetes	20.55	7	7357	1120	6472	106	242,726	369,856	127,236	1594	456	1138	1244
Zygosaccharomyces rouxii	Ascomycetes	9.76	7	5332	166	4991	65	123,372	154,232	353,232	4429	2634	1795	1860
TOTAL						210,928	5255	6,972,893	9,184,052	902,110	105,739		42,587	47,842

The first approach merely involved identifying ORFs from the existing available annotations for sequences that fitted the maximum 80 residues criterion used for this study. This approach was dependent on parameters had been set by the annotators of the deposited data as the minimum number of codons that were to be considered as protein coding. The sORFs retrieved from this extraction provided a reference for what had already been identified. The second approach, which can be considered as the major feature of this work, involved repeating the gene prediction and annotation process by specifically identifying potential ORFs in the intronic, exonic and intergenic regions. We had opted to focus the searches on sequences extracted from the intronic and intergenic regions because a relatively high number of sORFs can be found within these regions as demonstrated by the discoveries of 3241 putative sORFs in the intergenic regions of *Arabidopsis thaliana* [28] and 15 sORFs in the intronic regions of *Drosophila* [29]*.*

The sORF identification in the second approach involved the use of two computer programs, sORFfinder and getorf. The sORFfinder program was specifically designed to detect small ORFs. The getorf program, which is available as part of the emboss package, employs a less stringent approach that simply involves setting the sequence length parameter for genes to be below 240 nucleotides within the start to stop codons reading frame. In order to throw a wider net, we specifically included intronic and intergenic sequences as inputs for sORF identification. It is not unexpected that the output of both programs would contain false positives. In order to narrow down the selection, we had only selected outputs that were agreed on by both sORFfinder and our getorf runs at the 240 nt cap. This filtering was done in order to reduce the number of sequences for further characterization. It is however possible that true sORFs are present in the dataset that were predicted by only one of the programs and thus not investigated further as a part of this work. This is a clear limitation of the process that we had introduced as a means to acquire a more manageable number of sequences for further characterization.

The bypassing of sORFs that are located in the intergenic regions can occur during what is considered as the standard gene prediction process because these stretches of sequence only have sufficient length to encode for polypeptides that may be shorter than 100 residues [12, 27] and are thus overlooked as simply being non-coding filler sequences between two coding sequences. In order to address the possibility that a large number of sORFs in the intergenic regions may have been missed during a standard gene prediction process as demonstrated by the work of Hanada et al. that identified novel small open reading frames that were confirmed to at least be transcribed [28], our analysis also specifically targeted for the presence of sORFs in those sequences.

**Fig. 4:**
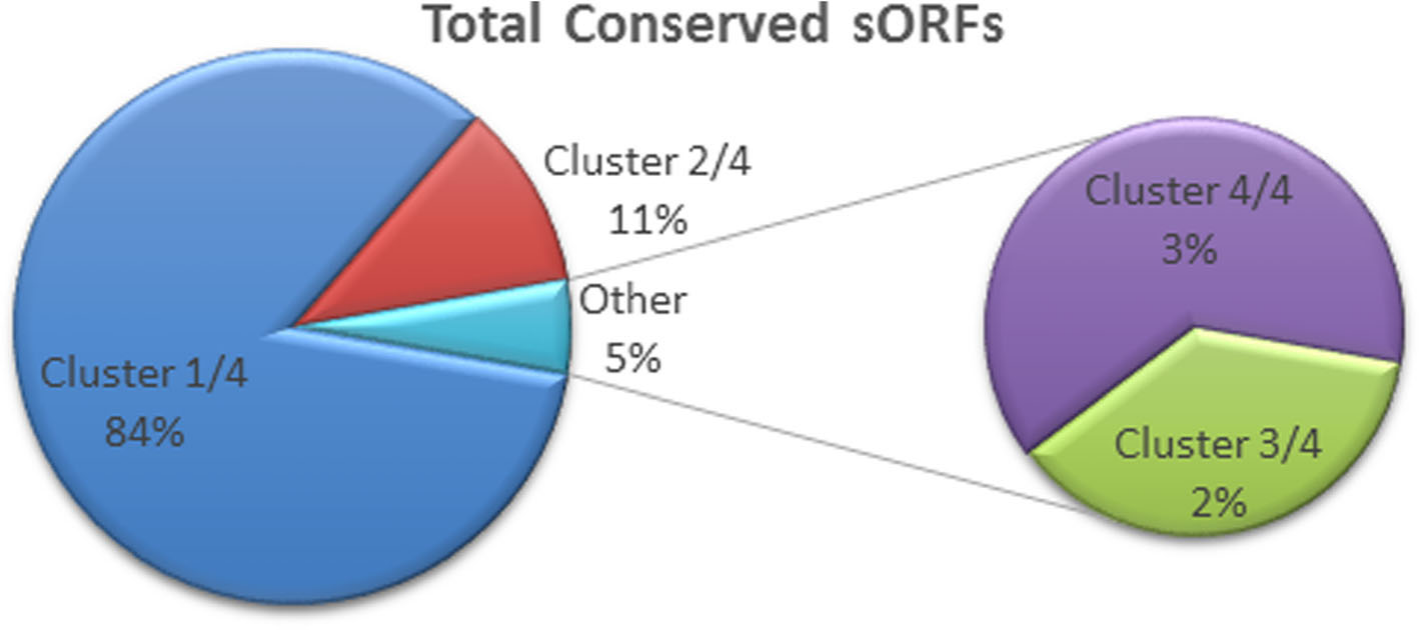
Clustered conserved sORFs

**Fig. 5:**
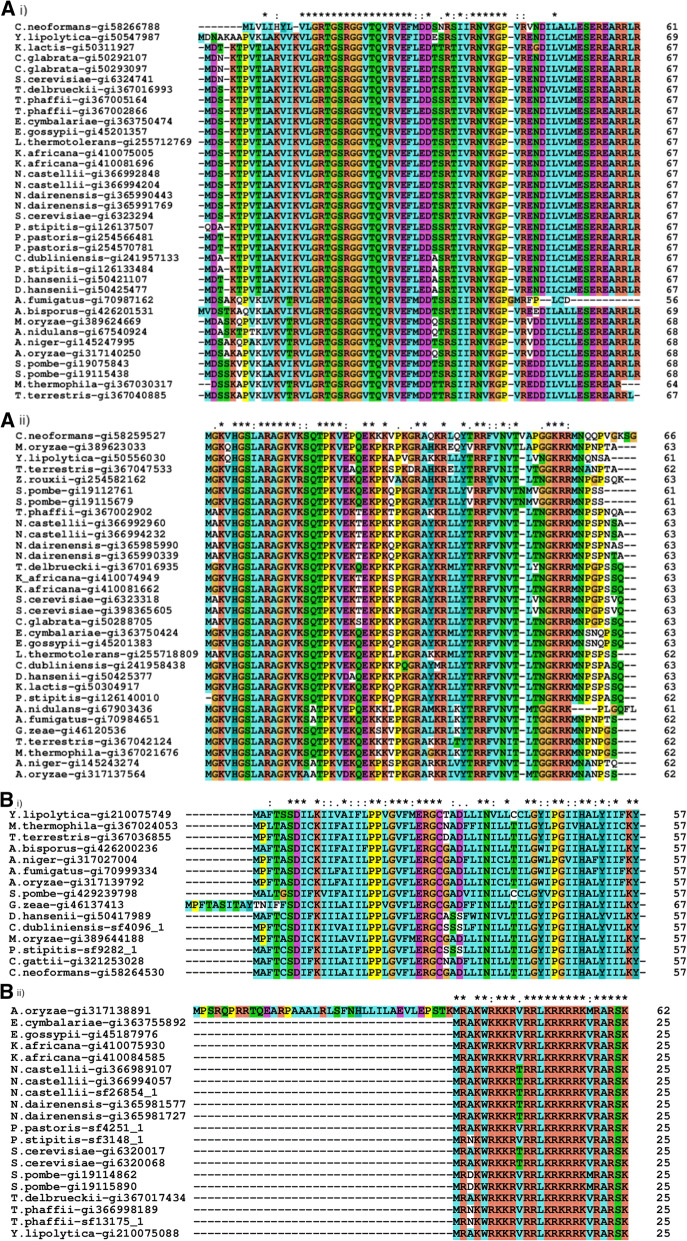
Multiple sequence alignments for sORFs that are conserved within (**a**) 26 fungal genomes (i-xx3497 and ii-xx4629) and (**b**) 2/4 fungal genomes (i-xx4249 and ii-xx6165) based on clustering. The sORFs extracted from genome annotations have identifiers with ‘*gi*’ while those computed from this work have identifiers with ‘*sf*’

**Fig. 6:**
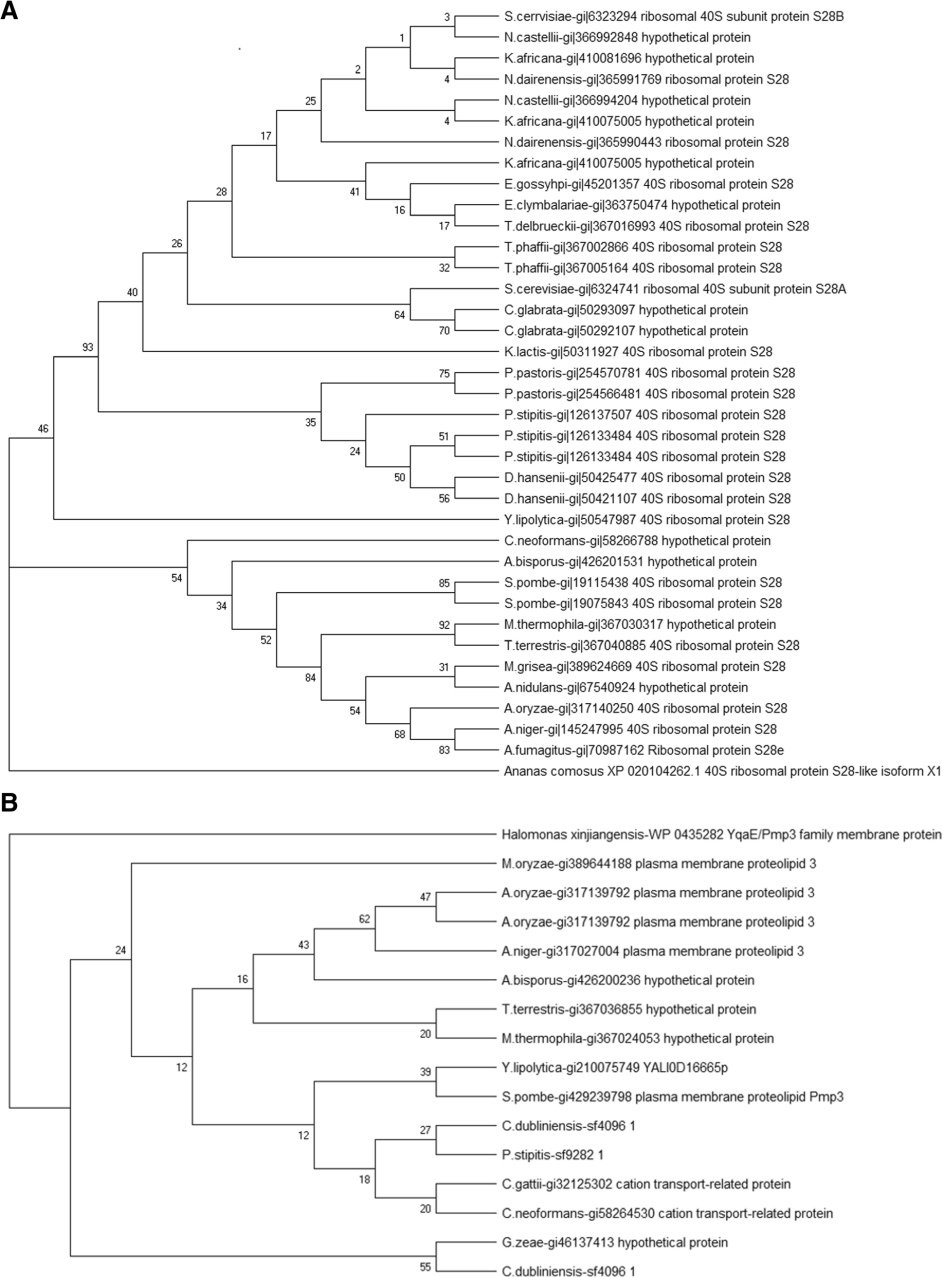
Phylogenetics of conserved sORFs within (**a**) 26 fungal genomes (xx3497) and (**b**) 2/4 fungal genomes (xx4249) based on clustering

Although there were no predicted sORFs that were conserved in all 31 genomes, there were 68 sORFs from two homologous clusters that were present in 26 of the 31 fungal genomes. Additionally, there are 1663, 215, and 40 sORFs that could be found in ¼, ½ and ¾ of the 31 genomes, respectively (Fig. 4). The two clusters identified by the genome screening approach consist of sORFs that are homologous to 40S ribosomal protein S28 (Fig. 5a i) and 40S ribosomal protein S30 (Fig. 5a ii). In the first cluster, 11 sORFs from eight species, *Cryptococcus neoformans, Candida glabrata, Eremothecium cymbalariae, Kazachstania africana, Naumovozyma castellii, Agaricus bisporus, Aspergillus nidulans* and *Myceliopthora thermophila* that were originally annotated as hypothetical proteins, were updated to be homologs of 40S ribosomal protein S28. The annotation for this homology assignment was obtained using BLAST and domain analysis using InterProScan. Furthermore, the evolutionary analysis on this cluster showed that all of these sORFs are conserved in fungi and are closely related in the fungal group when compared against the outgroup, *Ananas comosus* (pineapple) (Fig. 6a). This demonstrates the utility of reannotation projects in general and especially when they are designed to identify specific targets such as the one we have carried out in updating the existing annotation.

The function of sORFs in the first alignment set, which are conserved in about half of the 31 genomes (Fig. 5b i), are proteolipid membrane potential modulators that modulate the membrane potential, particularly to resist high cellular cation concentration. In eukaryotic organisms, stress-activated mitogen-activated protein kinases normally play crucial roles in transmitting environmental signals that will regulate gene expression for allowing the cell to adapt to cellular stress [30]. This protein is an evolutionarily conserved proteolipid in the plasma membrane which, in *S. pombe*, is transcriptionally regulated by the Spc1 stress MAPK (mitogen-activated protein kinases) pathway. There are two sORFs (*C. dubliniensis*-sf4096_1 and *P. pastoris*-sf9282_1) from the computational reannotation that were clustered together with conserved alignments, and thus indicating they may have the same function (Table 2). Further evolutionary analysis of this cluster showed that all of the sORFs in this cluster are closely related in fungal group against bacteria, *Halmonas xinjiangensis* (Fig. 6b).

**Table 2:** Characterization of sORFs conserved in 15 fungal genomes

sORFs ID	Access Number	Existed Description	New Description
A.bisporus-gi426200236	EKV50160.1	hypothetical protein AGABI2DRAFT_115218	plasma membrane proteolipid 3
A.fumigatus-gi70999334	XP_754386.1	stress response RCI peptide	plasma membrane proteolipid 3
A.niger-gi317027004	XP_001399936.2	plasma membrane proteolipid 3	plasma membrane proteolipid 3
A.oryzae-gi317139792	XP_003189201.1	plasma membrane proteolipid 3	plasma membrane proteolipid 3
C.dubliniensis-sf4096_1	NA	NA	plasma membrane proteolipid 3
C.gattii-gi321253028	XP_003192603.1	cation transport-related protein	plasma membrane proteolipid 3
C.neoformans-gi58264530	XP_569421.1	cation transport-related protein	plasma membrane proteolipid 3
D.hansenii-gi50417989	XP_457739.1	DEHA2C01320p	plasma membrane proteolipid 3
G.zeae-gi46137413	XP_390398.1	hypothetical protein FG10222.1	plasma membrane proteolipid 3
M.oryzae-gi389644188	XP_003719726.1	plasma membrane proteolipid 3	plasma membrane proteolipid 3
M.thermophila-gi367024053	XP_003661311.1	hypothetical protein MYCTH_2314489	plasma membrane proteolipid 3
P.stipitis-sf9282_1	NA	NA	plasma membrane proteolipid 3
S.pombe-gi429239798	NP_595350.2	plasma membrane proteolipid Pmp3	plasma membrane proteolipid 3
T.terrestris-gi367036855	XP_003648808.1	hypothetical protein THITE_2106674	plasma membrane proteolipid 3
Y.lipolytica-gi210075749	XP_502906.2	YALI0D16665p	plasma membrane proteolipid 3

The second alignment in Fig. 5b ii shows four sORFs predicted from the reannotation that are homologs to the 60S ribosomal protein. This is a possible indicator that sORFs may have been missed during a standard genome annotation process. Our analysis identified a higher number of sORFs candidates in *S. cerevisiae* compared to that published by Kastenmayer et al. [14]. The total of 77 sORFs predicted for *S. cerevisiae* contained all the 16 sORFs predicted by Kastenmayer et al. There are 20 sORFs in this set that were predicted by the sORFfinder and getorf integrated prediction process (Table 3). The other 57 sORFs predicted for *S. cerevisiae* have already been previously identified and was extracted from the genome screening approach.

**Table 3:** List of sORFs predicted in *S. cerevisiae*

from this study	Kastenmayer et al	from this study	Kastenmayer et al
S.cerevisiae-gi14318502	YFL017W-A	S.cerevisiae-gi6323292	#N/A
S.cerevisiae-gi398364355	YFR032C-A	S.cerevisiae-gi6323318	#N/A
S.cerevisiae-gi398365385	YNL024C-A	S.cerevisiae-gi6323506	#N/A
S.cerevisiae-gi398365605	YLR287C-A	S.cerevisiae-gi6323558	#N/A
S.cerevisiae-gi398365775	YOR210W	S.cerevisiae-gi6323634	#N/A
S.cerevisiae-gi398365789	YDR139C	S.cerevisiae-gi6323912	#N/A
S.cerevisiae-gi398366075	YLR388W	S.cerevisiae-gi6324184	#N/A
S.cerevisiae-gi6321622	YGR183C	S.cerevisiae-gi6324259	#N/A
S.cerevisiae-gi6321937	YHR143W-A	S.cerevisiae-gi6324313	#N/A
S.cerevisiae-gi6323294	YLR264W	S.cerevisiae-gi6324619	#N/A
S.cerevisiae-gi6323357	YLR325C	S.cerevisiae-gi6324877	#N/A
S.cerevisiae-gi6324070	YNL259C	S.cerevisiae-gi6325391	#N/A
S.cerevisiae-gi6324360	YNR032C-A	S.cerevisiae-gi73858744	#N/A
S.cerevisiae-gi6324741	YOR167C	S.cerevisiae-gi7839147	#N/A
S.cerevisiae-gi7839181	YHR072W-A	S.cerevisiae-sf1119_1	#N/A
S.cerevisiae-gi12621478	#N/A	S.cerevisiae-sf19568_1	#N/A
S.cerevisiae-gi147921768	#N/A	S.cerevisiae-sf21_1	#N/A
S.cerevisiae-gi33438768	#N/A	S.cerevisiae-sf21973_1	#N/A
S.cerevisiae-gi33438785	#N/A	S.cerevisiae-sf22173_1	#N/A
S.cerevisiae-gi33438820	#N/A	S.cerevisiae-sf23868_1	#N/A
S.cerevisiae-gi33438821	#N/A	S.cerevisiae-sf27242_1	#N/A
S.cerevisiae-gi33438834	#N/A	S.cerevisiae-sf27243_1	#N/A
S.cerevisiae-gi33438835	#N/A	S.cerevisiae-sf27714_1	#N/A
S.cerevisiae-gi33438838	#N/A	S.cerevisiae-sf3100_1	#N/A
S.cerevisiae-gi33438839	#N/A	S.cerevisiae-sf31758_1	#N/A
S.cerevisiae-gi398365465	#N/A	S.cerevisiae-sf32431_1	#N/A
S.cerevisiae-gi398365709	#N/A	S.cerevisiae-sf32615_1	#N/A
S.cerevisiae-gi398366109	#N/A	S.cerevisiae-sf34463_1	#N/A
S.cerevisiae-gi398366483	#N/A	S.cerevisiae-sf35098_1	#N/A
S.cerevisiae-gi398366543	#N/A	S.cerevisiae-sf4587_1	#N/A
S.cerevisiae-gi398366617	#N/A	S.cerevisiae-sf7880_1	#N/A
S.cerevisiae-gi41629681	#N/A	S.cerevisiae-sf85063_1	#N/A
S.cerevisiae-gi6226526	#N/A	S.cerevisiae-sf85096_1	#N/A
S.cerevisiae-gi6226533	#N/A	S.cerevisiae-sf9229_1	#N/A
S.cerevisiae-gi6320017	#N/A	S.cerevisiae-gi6320482	#N/A
S.cerevisiae-gi6320068	#N/A	S.cerevisiae-gi6320734	#N/A
S.cerevisiae-gi6320142	#N/A	S.cerevisiae-gi6320819	#N/A
S.cerevisiae-gi6320291	#N/A	S.cerevisiae-gi6322272	#N/A
S.cerevisiae-gi6323020	#N/A		

In 274 clusters predicted from the 31 fungal genomes, 892 putative conserved sORFs have been annotated previously as a gene and have known functions. Characterization of the putative conserved sORFs revealed that approximately 3.8% of the newly predicted sORFs have known functions but were not annotated as genes in the available genome annotations. Our sORF annotation workflow also determined that 832 of the putative conserved sORFs predicted are hypothetical proteins or have no characterized function (Fig. 3). Even though these sORFs do not have a known function, their conservation across multiple species imply that their presence is of some functional importance. Out of the total of 848 predicted sORFs from the 31 genomes (Fig. 3), 93 sORFs from the sORFfinder-getorf integration output have homologs in other organisms (Additional file 2).

**Fig. 7:**
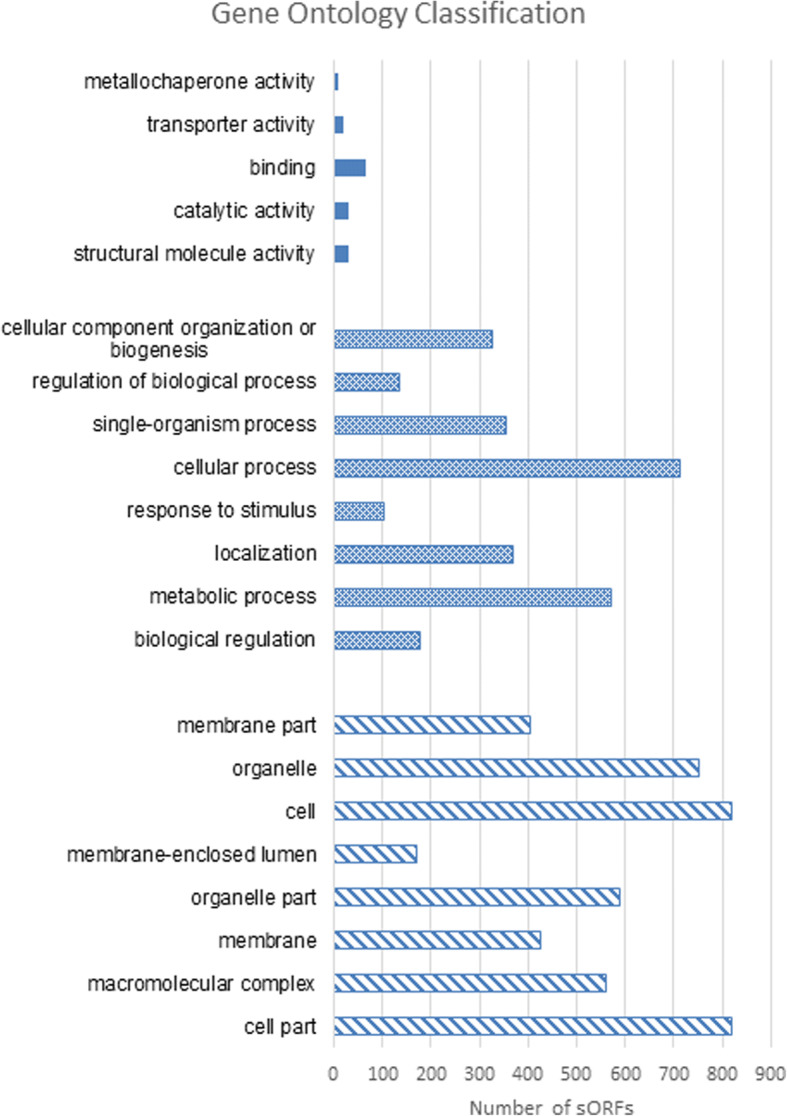
Classification of predicted conserved sORFs based on Gene Ontology. The solid colors represents cellular components, the dot patterns represents biological processes and cross patterns represents molecular functions

The total 1986 predicted sORFs were blast searched against the refseq database [31] and classified according to the three major Gene Ontology (GO) classes of molecular function, biological process and cellular component. Of the 1986 sORFs predicted, only 617 predicted sORFs could not be classified according to GO classes. This resulted in 2746 putative sORFs being classified into biological process, 4546 putative sORFs classified as cellular components and 155 predicted sORFs classified to be involved in molecular function (Fig. 7). The number of genes resulting from the Gene Ontology classification are higher than the total number of predicted sORFs predicted because one gene can be associated with multiple classes. The overall classification showed that most of the sORFs predicted have roles in biosynthesis and nucleic acid metabolism.

In the cellular component classification - there were 184 predicted sORFs classified into mitochondria (59), nucleus (57), endoplasmic reticulum (13), integral component of membrane (33) and Ssh1 translocon complex (22). Under the molecular function classification - the predicted sORFs were assigned to functions associated to mating pheromone activity (11), DNA and RNA binding (41), ribosome (145), cytochrome (7), protein binding (31), zinc ion binding (22), hydrogen ion transmembrane transporter activity (39), metal ion binding (16), protein heterodimerization activity (1), oxidoreductase activity (1), ATP binding (1), GTP binding (1) and ligase activity (1). For biological process GO classification, the 115 predicted sORFs in this group were classified into ribosome biogenesis (26), carbohydrate metabolic process (2), mitochondrial electron transport (4), DNA repair (1), mRNA export from nucleus (4), translation (10), protein N-linked glycosylation (1), protein targeting and targeting (3), copper ion transport (5), nucleocytoplasmic transport (14), response to stress (2), protein secretion (3), protein import into mitochondrial matrix (7), mitochondrial respiratory chain complex IV assembly (14), regulation of catalytic activity (8), transmembrane transport (1), vacuolar proton-transporting V-type ATPase complex assembly (5) and mitochondrial outer membrane translocase complex assembly (1).

Based on the cellular component classification, the secreted sORFs are associated with roles in communication, differentiation and establishing clonal behaviour. The secreted sORFs predicted that was associated to mating pheromone activity were 34–35 amino acids in length. There are 51 predicted sORFs that were associated with functions as membrane features or even in modulating cell membrane thickness or fluidity to respond to changing environmental conditions. One such example is the predicted sORF encoding 52 residues that is associated with plasma membrane proteolipid 3 (Pmp3p), which is part of the phosphoinositide-regulated stress sensor that has a role in the modulation of plasma membrane potential and in the regulation of intracellular ion homeostasis [32].

The methods that we have developed from available and proven tools are expected to be easily deployable to other genomes as and when they become available with minimal modifications. Recently, a psychrophilic yeast genome had been reported [33] that has other functional data also available such as gene expression during cold stress [34, 35] and the characterization of proteins involved in cold adaptation [36–38]. The mining of such genomes for sORFs that can then be integrated to the functional data may be a cost-effective means of identifying sORFs that are involved in psychrophily or other relevant extremophilic adaptations.

## Conclusions

The results of our work reveal that a high number of potential sORFs could be overlooked by the standard gene prediction workflow. We therefore recommend that the standard genome annotation process be complemented by analyses that specifically target the annotation of sORFs [39, 40], and then have both results integrated to provide a more complete genome annotation. This workflow is applicable for big data analysis because this study involved a large number of sequences from 31 completed fungal genomes that consisted of intergenics, introns, ORFs and genome sequences. Although the functional validation for predicted sORFs cannot be done based solely on the genome sequence without any corresponding transcriptomic or proteomic data, it is still possible to imply a putative status for the predicted sORFs by evaluating their conservation with the assumption, albeit a very simplistic one, that the observed conservation implies a conserved function of some biological importance and thus less likely to be artefacts of the gene prediction process. Furthermore, the predicted sORFs predicted will be incorporated into a database consisting of sORFs from fungal genomes.

## Methods

A workflow was created to predict sORFs from fungal genomes (Fig. 1) and the components and steps involved are provided below. The source code for the programs in this workflow have been deposited on GitHub - https://github.com/firdausraih/sORFs-fungal-genomes (Additional file 3). The data for sORFs were sourced from two datasets: (i) existing annotations made available with the genome sequences and (ii) a purpose built search.

### Source of genome data

The data for 31 fungi genomes were downloaded via FTP from the NCBI at ftp://ftp.ncbi.nlm.nih.gov/genomes/archive/old_refseq/Fungi/ (Table 1). These 31 fungi genomes were selected from 36 fungi genomes in NCBI based on the completeness of their genome analysis and annotation.

### Screening sORFs from genome annotation

Known or existing sORF annotations were first extracted from the existing genome annotations available for the fungal genomes used. This dataset was restricted to annotations for a maximum length of less than 240 nucleotides or 80 amino acids.

### Identification of intergenic, intronic and coding regions

The intronic and coding regions for the genomes were identified using Artemis [41] [https://www.sanger.ac.uk/science/tools/artemis] based on the chromosome, scaffold or contig sequences and the protein coding sequences for each genome. The intergenic regions were extracted from the genome annotations in the General Feature Format (GFF) format using a Perl script. The intergenic regions were extracted from both the forward and reverse strands.

### Identification of sORF using sORFfinder-getorf approaches

Gene predictions that specifically targeted the identification of sORFs were done by using sORFfinder-getorf approaches that combined two programs: getorf and sORFfinder. The prediction of the sORFs were carried out for each scaffold and/or chromosome. The sORFs predictions using getorf from the EMBOSS package [23, 42] were restricted to a maximum length of 240 nucleotides. Identification of sORFs by sORFfinder [24] was carried out using a 0.5 probability parameter. The results of sORFfinder, which by default is set at a maximum of 100 amino acids, were then filtered for output containing 80 amino acids in length.

### Determining existing homologs for the predicted sORFs

The predicted sORFs from getorf and sORFfinder search outputs for each fungi genome were combined and clustered using CD-HIT-EST [25, 43] and those with 100% identify were removed. Unique sequences that represented each cluster were then used as BLAST queries to search against a database of open reading frames (ORFs) for 31 fungal genomes using BLASTX [44, 45]. BLAST hits that aligned to less than two thirds of the query sequences and with less than 30% sequence identity were removed and the remainder were used as a potential sORFs dataset.

### Identification of conserved sORFs

The pre-annotated sORFs and those that were predicted as potential sORFs were then combined and clustered using CD-HIT at 70% identity to remove clusters that contained only a single sequence. For each cluster, sORFs that have homologs in at least two different species in one cluster were considered as potentially conserved sORFs. All conserved sORFs were identified their Kozak sequences using CPC2 [26].

### Multiple sequence alignments and evolutionary analysis

Identification of conserved sORFs in the clusters were carried out using the MUSCLE [46] sequence alignment program. A multiple sequence alignment generated using MUSCLE, which included one out group identified by PSI-BLAST [45], was used as input to construct a phylogenetic tree with 1000 bootstrap replications using the Jones-Taylor-Thornton (JTT) model based on the Neighbor joining method using PHYLIP 3.695 [47, 48].

### Function prediction and classification

The predicted sORFs were annotated using blast, interpro and classified using BLAST2GO into the three main Gene Ontology classes of molecular function, biological processes and cellular component [49–51].

## Additional files

Additional file 1: A listing of 1986 putative conserved sORFs predicted. This file contains a list of 1986 putative conserved sORFs predicted from all fungal genomes that can be viewed using Microsoft excel or text viewer. (TXT 151 kb)

Additional file 2: List of predicted sORFs with homologs. This file contains a list of sORFs predicted from all fungal genomes with their homologs that can be viewed using Microsoft excel or a text viewer. (TXT 118 kb)

Additional file 3: Pseudocode for sORFs workflow. This file contains a pseudocode for finding sORFs workflow using Linux environment using BASH, PYTHON and the Perl programming language. (ZIP 8 kb)

## Abbreviations

BLAST: Basic Local Alignment Search Tool
CD-HIT: Cluster Database at High Identity with Tolerance
EMBOSS: The European Molecular Biology Open Software Suite
FTP: File Transfer Protocol is a standard network protocol used for the transfer data
MUSCLE: MUltiple Sequence Comparison by Log-Expectation
NCBI: National Center for Biotechnology Information
ORFs: Open Reading Frames
PHYLIP: PHYLogeny Inference Package
PSI-BLAST: Position-Specific Iterated BLAST
sORFs: small Open Reading Frames
